# Endovenous laser ablation versus conventional surgery (ligation and stripping) for primary great saphenous varicose vein: a systematic review and meta-analysis

**DOI:** 10.1097/MS9.0000000000001095

**Published:** 2023-07-25

**Authors:** Oshan Shrestha, Sunil Basukala, Niranjan Thapa, Sagun Karki, Prashant Pant, Sushanta Paudel

**Affiliations:** a Department of Surgery, College of Medicine; b Department of Surgery, Nepalese Army Institute of Health Sciences, Kathmandu; c Department of Medicine, Star Hospital, Sanepa, Lalitpur, Nepal

**Keywords:** endovenous laser ablation, ligation, stripping, varicose vein

## Abstract

**Background:**

The great saphenous varicose vein was managed with high ligation and stripping conventionally, but with the development of minimally invasive surgical techniques like endovascular laser ablation (EVLA), they have become popular. This systematic review and meta-analysis of randomized controlled trials aim to compare the short-term and long-term outcomes of these two modalities on headings like procedural time, technical success, recovery time, recurrences, cost-effectiveness, and complications.

**Materials and methods:**

The protocol followed in this study was registered prospectively in the Registry of Systematic Reviews/Meta-analyses. Electronic databases were searched with appropriate search terms for relevant studies, and after their screening, data was extracted. The odds ratio was used for dichotomous data, and the mean difference or standardized mean difference was used for continuous variables.

**Results:**

This study identified 18 publications (10 randomized controlled trials) with a total of 1936 patients. There was no difference in procedural time, recovery time, recurrences at 1, 2, and 5 years, or clinical severity score. The surgery group had 4.35 times higher statistically significant odds of being technically successful at 2 years, while pooling data on bruising, hematoma, sensory disturbance, infection, and phlebitis showed that the EVLA group was less likely to develop postoperative complications.

**Conclusion:**

Technical failures were more common in the EVLA, whereas postoperative complications were more common in the surgery group. Both have comparable clinical effectiveness, and neither modality has clear superiority over the other. Parameters like cost-effectiveness must be assessed at the hospital level before choosing the right procedure for the patients.

## Introduction

Highlights

- The varicose vein was managed with high ligation and stripping conventionally, but modalities like endovascular laser ablation are becoming popular.
- There was no significant difference in procedural time, recovery time, recurrences at 1, 2, and 5 years, or clinical severity score.
- Technical failures were more common in the endovascular laser ablation, whereas postoperative complications were more common in the surgery group.
- Other parameters like cost-effectiveness needed to be assessed at the hospital level before choosing the best modality for a patient.

Varicose veins are a common disorder with an estimated prevalence of less than 1–73%, varying widely among different ages, sexes, races, and geographies^1^. While a large proportion of people may remain asymptomatic initially, the common symptoms encountered as the disease progresses are leg pain, swelling, heaviness, itching, and cramping in the calf, most of which improve with rest, leg elevation, and the use of compression stockings. More severe presentations include skin pigmentation, dermatitis, venous ulcer formation, cellulitis, superficial thrombophlebitis, and lipodermatosclerosis^2,3^. Varicose veins also cause significant deterioration in the quality of life, particularly at later stages of the disease, which improves after the management of the disease^4,5^. Conventionally, varicosity in the great saphenous vein was managed with high ligation and stripping, and with the advent of minimally invasive surgery, other techniques like endovascular laser ablation (EVLA) became popular^6^. EVLA and conventional surgery are both effective management options, but EVLA provides those benefits as any other minimally invasive surgery in terms of less postoperative pain and an early return to normal activity and work^7^. But the chances of reopening of the great saphenous vein and recurrences are also higher following EVLA^8^. The short-term and long-term outcomes of these two modalities were needed to be studied to provide a concrete base with the data to facilitate evidence based practice.

This systematic review and meta-analysis of randomized controlled trials compared the short-term and long-term outcomes of the EVLA and conventional surgery groups. This present study aimed to study outcomes like procedural time, technical success, recovery time, recurrences, cost-effectiveness, and postoperative complications.

## Materials and methods

This study is in line with the PRISMA guidelines^9^ (Supplemental Digital Content 1, http://links.lww.com/MS9/A195) and AMSTAR guidelines^10^ (Supplemental Digital Content 2, http://links.lww.com/MS9/A196).

### Protocol registration

The protocol followed in this systematic review and meta-analysis was registered prospectively in the Registry of Systematic Reviews/Meta-analyses^11^.

### Search strategy

Electronic databases (PubMed, PubMed Central, Scopus, and Embase) were searched with the use of terms like (‘endovenous laser ablation’), (EVLA), (ablation), (‘saphenofemoral ligation’), (‘saphenofemoral junction ligation’), (‘high ligation’), (‘varicose vein’) combined with appropriate Boolean operators. No time filters were used at the time of the electronic database search. Search details and the results of each database search are available as Supplementary File 1 (Supplemental Digital Content 3, http://links.lww.com/MS9/A197).

### Inclusion criteria and exclusion criteria

Randomized controlled trials comparing the outcomes of EVLA and ligation with stripping for great saphenous varicose vein were included in this study, while other study designs like cohort, nonrandomized studies, case–control studies, cross-sectional studies, editorials, and commentaries were excluded from the study.

### Study selection

Results of electronic database searches were handled with Covidence software^12^ and screening of the references was performed by two independent reviewers. A third reviewer resolved any conflicts that arose during screening. The roles were exchanged during the title and abstract screening and the full-text screening.

### Data curation

Data from the selected studies were extracted using a template prepared in Word. The template extracted the data under headings like study details, population, intervention, comparator, and outcome. Study details like the author’s list, the origin of the study, and the study period were extracted; under population, the demographic profile of the study group and baseline parameters were extracted; under intervention, the name of the intervention and similarly, under comparator, the procedure to which comparison is made was extracted. Outcomes such as procedural time, technical success, recovery time, recurrences, cost-effectiveness, and postoperative complications were extracted.

### Data synthesis

The odds ratio (OR) for dichotomous variables and the standardized mean difference or mean difference for continuous variables were used as effect measures. The *I*^2^ test was used to assess heterogeneity, and a fixed or random-effect model was used as per heterogeneity^13^. A fixed effect model is used for heterogeneity of up to 40% and beyond this, a random-effect model is used. Mean and SD are derived for studies reporting median and interquartile range by using the standard conversion formula^14^. The data obtained are expressed with a 95% CI. Forest plots are used to give visual feedback.

### Risk of bias assessment

**Figure 1:**
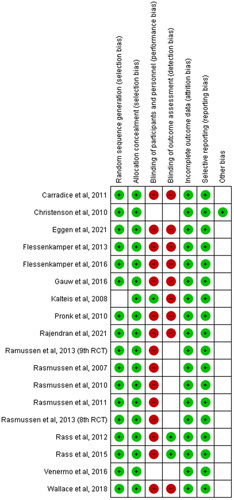
ROB of included studies.

The risk of bias assessment was carried out using the ROB tool. Two independent reviewers did the assessment and any disparity that was seen was solved by a third reviewer. The assessment of bias is shown in Figure 1.

### Sensitivity analysis

A sensitivity analysis was carried out for the results obtained by excluding each study at a time for every outcome.

## Results

**Figure 2:**
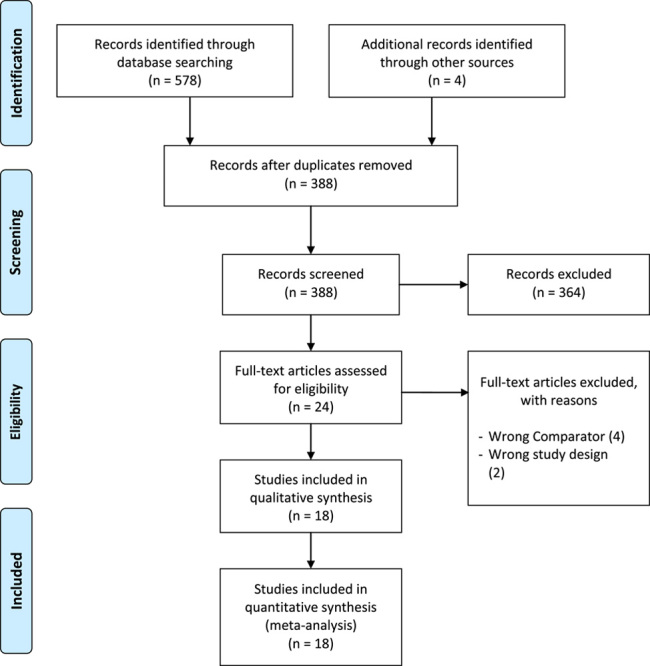
PRISMA flow diagram.

This study of 10 randomized controlled trials, whose outcomes were published in 18 different studies, involved a total of 1936 patients. The search of databases yielded 578 studies, and an additional 4 studies were added from other sources. After the removal of duplicates, 388 studies were screened. After the screening, 18 studies were identified as matching the inclusion and exclusion criteria of this study. Details are shown in Figure 2.

### Qualitative synthesis

This study included nine randomized controlled trials, and studies that reported the outcomes of the same randomized controlled trials are shown in the same section. A summary of the details of the included studies is shown in Table 1.

**Table 1:** Summary of included studies

RCT number	Study ID	Population	Intervention	Comparison	Outcome
1	Carradice *et al*.^7^	*N*=276 (T=139, C=137) Male T=54/139, C=47/137 Female T=85/139, C=90/137 Value (Mean±SD), Age: T=49±14, C=49±13 Antiplatelets/anticoagulants: T=9/139, C=12/137 Height (m): T=1·7±0·1, C=1·7±0·1 BMI (kg/m^2^): T=26·6±5·0, C=26·0±4·3 GSV diameter (mm), Groin: T=8·7±2·8, C=8·2±2·7 Knee: T=6·7±1·8, C=6·7±2·0 Venous Clinical Severity Score: T=4 (3–5), C=4 (3–5)	EVLA	SFJ ligation and stripping	Postoperative Complications Sensory disturbance: T=4/137, C=13/133 Hematoma: T=1/137, C=11/133 Infection: T=2/137, C=8/133 Phlebitis: T=4/137, C=6/133 Persistent pain: T=1/137, C=5/133 Pigmentation: T=4/137, C=1/133 Anesthetic complication: T=0, C=3/133 Persistent bruising: T=1/137, C= 2/133 Allergy: T=0, C=1/133 Procedural time (minutes): T=67±16, C=61±14 Return to normal activities (days): T=4.25±2.62, C=15±5.20 Return to work (days): T=6±3.49, C=17.25±4.36 In 3 months VCSS: T=0.75±0.14, C=0.75±0.14 Aberdeen Varicose Vein Questionnaire scores: T=2.6±1.8, C=3±2.02 Cosmetic satisfaction: T=9.2 (8–10), C=9 (8–10) Overall satisfaction: T=10 (9.5–10), C=10 (9–10)
	Wallace *et al*.^15^				In 1 year VCSS: T=0.75±0.14, C=0.75±0.14 Aberdeen Varicose Vein Questionnaire scores: T=2.3±1.5, C=2.3±1.6 Cosmetic satisfaction: T=9.8 (9–10), C=9.20 (8–10) Overall satisfaction: T=10 (9.5–10), C=10 (9.10–10) In 2 years VCSS: T=0.25±0.38, C=0.25±0.38 Aberdeen Varicose Vein Questionnaire scores: T=2.54±1.78, C=3.7±2.04 Cosmetic satisfaction: T=10 (8–10), C=9 (8–10) Overall satisfaction: T=10 (9.6–10), C=10 (9–10) Recurrence: T=19/139, C=37/137 In 5 years VCSS: T=0.25±0.38, C=1±0.61 Aberdeen Varicose Vein Questionnaire scores: T=3.35±1.85, C=4.88±2.67 Cosmetic satisfaction: T=10 (8–10), C=9 (7–10) Overall satisfaction: T=10 (9–10), C=10 (9–10) Recurrence: T=29/139, C=47/137
2	Christenson *et al*.^8^	*N*=200 (T=100, C=100) Male T=33/100, C=29/100 Female T=67/100, C=71/100 Value (Mean±SD), Age: T=44.6±10.5, C1=46.3±13.3 BMI (kg/m^2^): T=26.2±4.8, C=26.0±5.1 Size of GSV at 3 cm from SFJ (mm): T=6.9±2.0, C=6.6±1.7 Reflux time (sec): T=2.5±0.9, C=2.4±1.1 Ankle-brachial index: T=0.9±0.1, C=0.9±0.1 Varicose Vein Clinical Severity Score: T=5.2±2.5, C=5.2±2.7 Aberdeen Varicose Vein Severity Score: T=22.5±6.5, C=22.0±7.5	EVLA	SFJ ligation and stripping	At 12th day GSV absent or abolished: T=100/100, C=100/100 Detectable reflux: T=1/100, C=0 Symptoms: T=2/100, C=18/100 Superficial localized phlebitis: T=4/100, C=1/100 Deep vein thrombosis: T=1/100, C=0 Hematoma: T=5/100, C=12/100 Transient paresthesia: T=1/100, C=1/100 Bruising: T=1/100, C=2/100 At 1 year Varicose Vein Clinical Severity Score: T=0.26±0.68, C=0.23±0.57 Aberdeen Varicose Vein Severity Score: T=4.53±3.10, C=4.17±1.97 At 2 Years Great saphenous vein Absent/completely closed: T=88/95, C=99/99 Technical failure: 7/95, C=0 Completely reopened: T=2/95, C=0 Partially reopened: T=5/95, C=0 Reflux, No: T=8/95, C=2/99 Limbs with symptoms, No: T=9/95, C=1/99 Limbs re-operated on, No: T=3/95, C= 0 Varicose Vein Clinical Severity Score: T=0.23±0.54, C=0.23±0.59 Aberdeen Varicose Vein Severity Score: T=3.82±1.35, C=3.54±2.30
3	Pronk *et al*.^16^	*N*=130 (T=62, C=68) Male T=16/62, C=46/68 Female T=15/62, C=53/68 Value (Mean±SD), Age: T=49±11.0, C=50±10.5 BMI: T=25±3.3, C=24.5±3.7 Diameter of SFJ (cm): T=0.88±0.22, C=0.92±0.27 Diameter GSV (cm): T=0.64±0.16, C=0.64±0.14 Preoperative complaints, Tired legs: T=31/62, C=35/68 edema: T=21/62, C=32/68 Itching: T=20/62, C=26/68 Cosmetic: T=13/62, C=13/68 Pain: T=9/62, C=13/68 Restless legs: T=11/62, C=6/68 Calf cramps: T=8/62, C=8/68 Other: T=9/62, C=7/68	EVLA	SFJ ligation and stripping	Restart normal activities (days): T=3.16±4.34, C=3.20±4.01 Restart work (days): T=4.38±5.43, C=4.15±3.72 Restart sport (days): T=10.52±7.12, C=10.62±6.96 Pain scale, Day 1: T=3.58±2.60, C=4.00±2.34 Day 14: T=1.66±2.04, C=0.77±1.46 At 1 year Total recurrence: T=5/56, C=5/49 Clinical recurrence: T=3/49, C=3/56 Tired legs: T=5/56, C=8/68 Edema: T=6/56, C=10/68 Itching: T=3/56, C=6/68 Cosmetic: T=4/56, C=8/68 Pain: T=1/56, C=6/68 Restless legs: T=7/56, C=4/68 Calf cramps: T=5/56 C=2/68 Other: T=2/56, C=1/68
	Gauw *et al*.^17^				At 5 years Total recurrence: T=33/61, C=12/60 Clinical recurrence: T=20/61, C=10/60 Reintervention: T=11/61, C=7/60 Tired legs: T=1/61, C=1/60 Edema: T=6/61, C=6/60 Itching: T=3/61, C=1/60 Cosmetic: T=8/61, C=3/60 Pain: T=4/61, C=1/60 Restless legs: T=4/61, C=0 Calf cramps: T=7/61, C=2/60 Other: T=2/61, C=1/60 Persisting neurosensory deficit: T=0, C=1/60
	Eggen *et al*.^18^				At 10 years Total recurrence: T=33/50, C=16/53 Clinical recurrence: T=25/50, C=14/53 Reintervention: T=18/50, C=9/53 Tired legs: T=1/50, C=0 Edema: T=1/50, C=1/53 Itching: T=1/50, C=3/53 Cosmetic: T=0, C=0 Pain: T=1/50, C=0 Restless legs: T=1/50, C=1/53 Calf cramps: T=0, C=0 Other: T=1/50, C=0 Persisting neurosensory deficit: T=1/50, C=0
4	Flessenkamper *et al*.^19^	*N*=301 (T=142, C=159) Male T=45/142, C=47/159 Female T=97/142, C=112/159 Value (Mean±SD), Age: T=47.7±12.9, C=47.7±11.5 Preoperative pain, None: T=73/142, C=98/159 Moderate (No analgesics): T=68/142, C=59/159 Severe (Analgescis): T=0, C=0 Lipodermatosis, Localized: T=10/142, C=17/159 Extended: T=0, C=1/159	EVLA	SFJ ligation and stripping	Postoperative evaluation Postoperative pain: T=50/142, C=51/159 Ecchymosis: T=68/142, C=108/159 Neurological deficits in the saphenous region: T=5/142, C=1/159 At 2 months Recurrence: T=38/142, C=0 Complications, Hyperpigmentation: T=18/142, C=11/159 Matting: T= 4/142, C=2/159 Edema: T=22/142, C=8/159 Lipodermatosis: T=10/142, C=12/159
	Flessenkamper *et al*.^20^				At 2 years Recurrence: T=20/112, C=11/94 At 3 years: Recurrence: T=12/68, C=12/63 At 4 years: Recurrence: T=15/61, C=10/56 At 5 years: Recurrence: T=11/45, C=14/53 At 6 years: Recurrence: T=15/38, C=15/43
5	Kalteis *et al*.^21^	*N*=95 (T=47 C=48) Male T=10/47, C=14/48 Female T=37/47, C=34/48 Value [Median (IQR)], Age: T=46 (38–57), C=46.5 (39–53) GSV diameter (cm): T=0.80 (0.7–1.0), C=0.80 (0.7–1.1) BMI: T=26.6 (23.7–30.4), C=25.3 (23.5–27.7) Venous clinical severity score: T=4.24, C=4.82 Pain (VAS scale), cm: T=0.4, C=0.8 Swelling: T=24/47, C=28/48 Heaviness: T=29/47, C=36/48 Cramps: T=18/47, C=11/48 Chronic Venous Insufficiency Questionnaire: T=84, C=77	EVLA	SFJ ligation and stripping	At 1 week Successful GSV treatment postoperative: (T=45/47, C=47/48) Paresthesia after surgery: T=16/47, C=21/48) VAS value [median (quartiles)]: T=2.13 (1.17–3.61), C=2.52 (1.24–4.19) Use of analgesics (mg/day) [median (quartiles)]: T=14.29 (7.14–35.71), C=17.86 (7.14–33.93) At 4 weeks Successful GSV treatment postoperative: (T=47/47, C=48/48) Paresthesia after surgery: (T=9/47, C=15/48) Residual hematomas, postoperative: (T=16/47, C=28/48) Cosmetic result, patient rating score: T=1.52±0.68, C=1.83±1.10 (1=good, 5=worst) At 16 weeks Successful GSV treatment postoperative: (T=47/47, C=48/48) Paresthesia after surgery: T=23/47, C=23/48) VAS value [median (quartiles)]: T=0.51 (0.14–1.21), C=0.55 (0.10–1.62) Residual hematomas, postoperative: (T=6/47, C=5/48) Cosmetic result, patient rating score: T=1.52±0.65, C=1.72±0.98 (1=good, 5=worst) Time to resume work (days) [Mean±SD]: T=19.87±3.32, C=16.45±3.55 Compression stocking use [Median (IQR)] (days) : T=42.00 (28.0–88.0), C=75.00 (58.0–105.0) Analgesics total use [Median (IQR)] (days): T=2.00 (1.00–5.00), C=2.00 (1.00–7.25)
6	Rajendran *et al*.^22^	*N*=80 (T=40, C=40) Male T=18/40, C=14/40 Female T=22/40, C=26/40 Value (Mean±SD), Age: T=46.8±13.1, C=46.7±11.5 GSV diameter (cm): T=5.4±1.0, C=7.0±2.3 Venous clinical severity score: T=9.3±3.2, C=9.2±3.7	EVLA	SFJ ligation and stripping	VAS score for pain At 8 h T=2.9±1.0, C=3.7±1.2 At 7 days T=1.8±0.7, C=1.4±0.5 At 1 month GSV recurrence: T=0, C=0 VCSS: T=3.9±1.5, C=5±1.7 At 6 months GSV recurrence: T=1/40, C=0 VCSS: T=2.1±0.8, C=2.4±1.3 At 1 year GSV recurrence: T=1/40, C=0 VCSS: T=1.3±0.6, C=1.3±0.7
7	Rass *et al*.^23^	*N*=346 (T=185, C=161) Male T=61/185, C=48/161 Female T=124/185, C=113/161 Value (Mean±SD), Age: T=47.9±10.9, C=48.0±10.7 BMI: T=26.2±4.1, C=26.3±4.9 Homburg Varicose Vein Severity Score (0–33): T=13.0±4.8, C=12.6±4.3 GSV diameter at SFJ (mm): T=8.7±2.8, C=8.7±2.2	EVLA	SFJ ligation and stripping	At 3 months Dysesthesia: T=17±9, C=22±14 Dyspigmentation: T=57±32, C=19±12 Homburg Varicose Vein Severity Score: T=3.9±3, C=3.8±3 At 1 year Dysesthesia: T=11±6, C=11±8 Dyspigmentation: T=31±18, C=8±6 Homburg Varicose Vein Severity Score: T=2.0±2, C=2.1±3 At 2 years Dysesthesia: T=7±4, C=11±8 Dyspigmentation: T=12±7, C=4±3 Homburg Varicose Vein Severity Score: T=2.1±3, C=1.9±3 Recurrence after surgery: T=28/173, C=33/143 Follow-up [median (range)] (months): T=24.4 (13.7–45.9), C=24.7 (20.5–47.7) Technical failure: T=6/28, C=2/33 VAS score (Mean±SD): T=1.6±0.8, C=1.3±0.6 (1–5 range) Pain duration (Mean±SD) (days): T=8±6, C=17±20
	Rass *et al*.^24^				At 5 years Recurrence after surgery: T=69/152, C=70/129 Follow-up [median (range)] (months): T=60.4 (51.6±79.2), C=60.7 (48.7±83.5) Persisting or recurrent reflux: T=27/69, C=0 Homburg Varicose Vein Severity Score: T=3.00±2.87, C=3.16±3.48 Overall satisfaction: T=1.28±0.51, C=1.39±0.58 Cosmetic score: T=1.59±0.78, C=1.72±0.91 Dysesthesia: T=4/152, C=2/129 Hyperpigmentation: T=0 C=1/129
8	Rasmussen *et al*.^25^	*N*=121 (T=62, C=59) Legs=137 (T=69, C=68) Male T=21/62, C=16/59 Female T=41/62, C=43/59 Value (Mean±SD), Age: T=53 (26–79), C=54 (22–78) Great saphenous vein. Diameter (mm): T=7.9±2.7, C=7.6±2.1 Reflux time (s): T=2.6±1.1, C=2.5±1.0	EVLA	SFJ ligation and stripping	Time to resume normal activity (days): T=6.9±7.0, C=7.7±6.1 Time to resume work (days): T=7.0±6.0, C=7.6±4.9 Total costs (euro): T=3396.4, C=3084.5 At 12 days Infection: T=0, C=1/59 Phlebitis: T=2/62, C=2/59 Bruising: T=7/62, C=15/59 Hematoma: T=3/62, C=5/59 Aberdeen Varicose Vein Symptoms Severity Score [Mean (range)]: T=23.1 (0–49.9), C=21.5 (0–42.6) At 1 month Phlebitis: T=2/62, C=2/59 Hematoma: T=0, C=1/59 Paresthesia: T=1/62, C=0 Aberdeen Varicose Vein Symptoms Severity Score [Mean (range)]: T=14.2 (0–47.9), C=13.7 (0–47.4) At 3 months Aberdeen Varicose Vein Symptoms Severity Score (Mean±SD): T=14.4±12.6, C=11.9±9.02 Venous Clinical Severity Score (Mean±SD): T=0.55±0.62, C=0.6±0.62 At 6 months Paresthesia: T=0, C=1/59 Aberdeen Varicose Vein Symptoms Severity Score [Mean (range)]: T=7.1 (0–38.7), C=5.3 (0–33.1) Venous Clinical Severity Score [Mean (range)]: T=0.4 (0–7), C=0.2 (0–2)
	Rasmussen *et al*.^26^				At 2 years Recurrence: T=18/69, C=25/68 Technical failure: T=3/69, C=2/68
	Rasmussen *et al*. (8th RCT)^27^				At 5 years Clinical recurrence: T=25/69, C=24/68 Technical failure: T=3/69, C=2/68 Retreatment: 17/69, C=15/68 Aberdeen Varicose Vein Symptoms Severity Score (Mean±SD): T=3.0±5.3, C=3.6±4.1 Venous Clinical Severity Score (Mean±SD): T=0.4±0.9, C=0.4±0.7
9	Rasmussen *et al*.^28^	*N*=249 (T=125, C=124) Legs=137 (T=144, C=142) Male T=35/125, C=29/124 Female T=90/125, C=95/124 Value [Median (IQR)], Age: T=52 (18–74), C=50 (19–72) GSV diameter (mm): T=7·6 (3–12), C=7·8 (3–14)	EVLA	SFJ ligation and stripping	Procedural time (Mean±SD) (minutes): T=61±48.52, C=39.75±18.78 Time to resume normal activity (Mean±SD) (days): T=7.25±7.25, C=9.5±8.68 Time to resume work (Mean±SD) (days): T=13.3±13.3, C=12.65±12.15 Total costs (euro): T=2200, C=2199 At 1 month Treatment Failure: T=1/144, C=3/142 Deep vein thrombosis: T=0, C=1/142 Phlebitis: T=4/144, C=5/142 Infection: T=0, C=1/142 Paresthesia: T=3/144, C=5/142 Hyperpigmentation: T=3/144, C=6/142 Hemorrhage: T=1/144, C=1/142 At 1 year Recurrence: T=14/144, C=16/142
	Ramussen *et al*. (9th RCT)^29^				At 3 years Recurrence: T=24/144, C=22/142 Reoperation: T=14/144, C=18/142 Aberdeen Varicose Vein Symptoms Severity Score (Mean±SD): T=4.61±5.8, C=4.00±4.87 Venous Clinical Severity Score (Mean±SD): T=0.34±1.3, C=0.3±0.5
10	Venermo *et al*.^30^	*N*=138 (T=73, C=65) Male T=18/73, C=10/65 Female T=55/73, C=55/65 Value (Mean±SD), Age: T=47.0±13.4, C=47.3±11.3 GSV diameter at SFJ (mm): T=6.3±1.1, C=6.2±1.1 Aberdeen Varicose Vein Severity Score: T=32.4±6.7, C=30.2±6.3 BMI: T=25.2±3.6, C=25.1±3.7	EVLA	SFJ ligation and stripping	Time to resume work (days): T=8±5, C=12±6 Complications Wound infection: T=3/73, C=3/61 Hematoma: T=30/73, C=38/61 Skin pigmentation: T=3/73, C=3/61 Paresthesia: T=1/73, C=2/61 Lumps in veins: T=34/73, C=33/61 At 1 year Technical failure: T=2/73, C=2/61 Total Recurrences: T=12/73, C=8/61

EVLA, endovenous laser ablation; GSV, greater saphenous vein; RCT, randomized controlled trial; SFJ, saphenofemoral junction; VCSS, venous clinical severity score.

### Quantitative synthesis

#### Procedural duration

**Figure 3:**
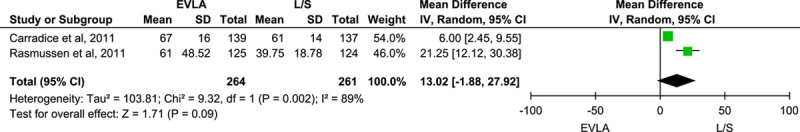
Forest plot for procedural duration outcome. EVLA, endovascular laser ablation.

Pooling data from two trials using a random-effect model for the duration of the procedure showed that ligation and stripping took 13.02 min less time compared to EVLA on average, but the result was not statistically significant (MD: 13.02; 95% CI: −1.88 to 27.92; *n*=525; *I*^2^=89%; *P*-value=0.09) (Fig. 3). On rerunning the analysis using the fixed effect model, it showed that ligation and stripping took 8 min lesser time to complete and was statistically significant (Fig. A, Supplementary File 2, Supplemental Digital Content 4, http://links.lww.com/MS9/A198).

#### Return to normal activities and work

**Figure 4:**
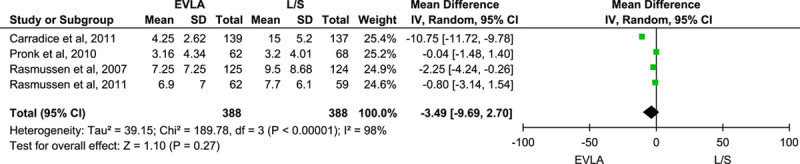
Forest plot for return to normal activities outcome. EVLA, endovascular laser ablation.

**Figure 5:**
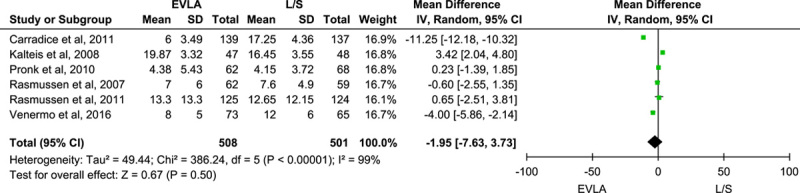
Forest plot for return to work outcome. EVLA, endovascular laser ablation.

Five trials reported the time taken to return to normal activities, and pooling of the data using the random-effect model did not show a statistically significant difference between the two groups (MD: −3.49; 95% CI: −9.69 to –2.70; *n*=776; *I*^2^=98%; *P*-value=0.27) (Fig. 4). Also, pooling data from six trials reporting the time taken to return to work outcome using the random-effect model did not show a statistically significant result (MD: −1.95; 95% CI: −7.63 to 3.73; *n*=1009; *I*^2^=99%; *P*-value=0.50) (Fig. 5). A sensitivity analysis done by excluding each study at a time showed no significant change. However, both of the outcomes showed statistically significant result on pooling the data using the fixed effect model. (Figs B and C, Supplementary File 2, Supplemental Digital Content 4, http://links.lww.com/MS9/A198).

#### Technical failure at 2 years

**Figure 6:**
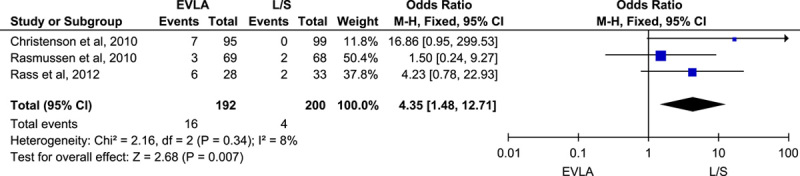
Forest plot for technical failure at 2 years outcome. EVLA, endovascular laser ablation.

Pooling the data from three trials reporting technical failure outcomes at 2 years by using the fixed effect model showed that the ligation and stripping group had 4.35 times more odds of remaining technically successful (OR: 4.35; 95% CI: 1.48–12.71; *n*=392; *I*^2^=8%, *P*-value=0.007) (Fig. 6). Sensitivity analysis excluding one study (Rasmussen *et al*. 2010) showed a significant rise in the odds of ligation and stripping being technically successful (OR: 7.24; 95% CI: 1.75–29.99; *n*=255; *I*^2^=0%, *P*-value=0.006) (Fig. D, Supplementary File 2, Supplemental Digital Content 4, http://links.lww.com/MS9/A198).

#### Recurrences at 1 year, 2 years, and 5 years

When the data on recurrence at 1 year was pooled from three trials, it showed that the difference between the two groups was not statistically significant. Also, pooling data on recurrence at 2 and 5 years showed no significant difference between the two groups. Details are provided in Supplementary File 3 (Supplemental Digital Content 5, http://links.lww.com/MS9/A199). Sensitivity analysis of results of 1 and 2 years showed no significant difference; however, sensitivity analysis of the result of 5 years by excluding one study (Gauw *et al*. 2016) significantly favored EVLA (OR: 0.70; 95% CI: 0.52–0.94; *n*=792; *I*^2^=8%, *P*-value=0.007) (Fig. E, Supplementary File 2, Supplemental Digital Content 4, http://links.lww.com/MS9/A198).

#### Clinical severity score

Three trials reported the varicose vein clinical severity score at one year, and pooling of those trials using the mean difference effect measure showed no significant difference. Also, three trials reported the data of clinical severity scores at 5 years and pooling of which by using a standardized mean difference showed no significant difference. Details and forest plots are given in Supplementary File 3 (Supplemental Digital Content 5, http://links.lww.com/MS9/A199). A sensitivity analysis of both results by excluding each study at a time showed no significant changes.

#### Complications

Pooling data on bruising, hematoma, sensory disturbance, infection, and phlebitis showed that the EVLA group was less likely to develop postoperative complications compared to the ligation and stripping group. The results were statistically significant except for sensory disturbance and phlebitis outcomes. Details are shown in Table 2 and Supplementary File 3 (Supplemental Digital Content 5, http://links.lww.com/MS9/A199).

**Table 2:** Postoperative complications

Outcomes	Effect measure	Effect model	Heterogeneity	Significance
Bruising	OR: 0.43; 95% CI: 0.28–0.64	Fixed	0%	<0.0001
Hematoma	OR: 0.35; 95% CI: 0.21–0.59	Fixed	0%	<0.0001
Sensory disturbance	OR: 0.63; 95% CI: 0.37–1.07	Fixed	35%	0.09
Infection	OR: 0.38; 95% CI: 0.14–1.01	Fixed	0%	0.05
Phlebitis	OR: 0.98; 95% CI: 0.46–2.07	Fixed	0%	0.95

OR: odds ratio

### Publication bias

Publication bias was assessed with the use of funnel plots. The funnel plot showed a symmetrical distribution for recurrence at 1 year, postoperative complications, and technical failure. Other outcomes (procedural time, recurrence at 2 and 5 years, return to normal activities and work, and clinical severity score) showed an asymmetrical distribution of studies depicting significant publication bias. Funnel plots are shown in Supplementary File 2 (Supplemental Digital Content 4, http://links.lww.com/MS9/A198).

## Discussion

The EVLA procedure includes the use of a laser, a form of electromagnetic energy, to obtain thermal ablation of the affected vein. Venous closure is achieved by heat-induced shrinkage of the collagen and fibrotic sealing of the lumen of the veins. The vein wall needs to absorb enough energy for the generation of enough heat to get obliterated^31^. In conventional surgery, it includes ligation of the saphenofemoral junction along with stripping of the affected veins. This study compared the outcomes of the patients treated by these two methods by including results from ten randomized controlled trials.

EVLA, being a newer, less invasive procedure that can be performed under local anesthesia, was thought to have clear-cut superiority to conventional surgery, but the results obtained did not exhibit such superior traits to vouch for EVLA and discard conventional surgery. This study found that the time taken for completion of the procedure between the two groups had no significant difference. Also, the time taken to return to normal activities and the time taken to return to work did not have a significant difference. Although EVLA is performed under local tumescent anesthesia, it did not have a statistically significant faster recovery time. Faster recovery time is one of the main reasons for preferring minimally invasive procedures, but EVLA showed no such merits over conventional surgery.

Three trials reported the technical failure outcome measured at 2 years, and a meta-analysis of the outcome showed that the ligation and stripping group was four times more likely to be technically successful compared to EVLA. The main reason for technical failure in the EVLA group is early recanalisation^26^. As EVLA works on the principle of achieving vein obliteration by letting the veins absorb enough energy for heat generation, the energy used during the procedure affects the outcome. A study showed that the energy delivered had a direct effect on recurrence, with the worst results on low-energy delivery compared to high-energy delivery^32^. Early recanalization is avoided when an energy of more than 80 joules per cm is used in the EVLA procedure^33^. Technical failure observed in the surgery group is due to misjudgement of the source of reflux (when USG-guided marking is not done preoperatively) and the breakage of the great saphenous vein during stripping or inadequate stripping^26^. Recurrence outcome was also studied, and it showed no difference between the two groups. Recurrences developing at 1 year, 2 years, and 5 years had no statistically significant difference. Only one of the included studies had follow-up results up to 10 years, which showed that total recurrence was 66% in the EVLA group and 30.18% in the surgery group, with 36% of the patients in the EVLA group and 16.98% of the patients in the surgery group needing reintervention^18^. Recurrence in the EVLA group is mainly attributed to technical failure and reflux into the anterior accessory greater saphenous vein, whereas recurrence in the surgery group is attributed to neo-vascularization and technical errors^34,35^.

Data from clinical severity score outcomes that assessed the severity of the disease on headings like pain, varicose vein, venous edema, skin pigmentation, inflammation, induration, number of active ulcers, ulcer duration, active ulcer size, and compression therapy^36^ was pooled and analyzed. It showed that clinical severity scores at 1 year and 5 years showed no significant difference between the two groups. Postoperative complications like bruising, hematoma, sensory disturbance, infection, and phlebitis were analyzed. This study found that the surgery group had statistically significantly higher complication rates compared to the EVLA group. This study also aimed to analyze and compare the cost of care, but due to the paucity of data, it could not be performed. However, two studies reported the cost of care, and they showed that the EVLA group had a higher cost of care compared to the surgery group. A cost-effectiveness study concluded that surgical treatment offered robust health benefits at a relatively lower cost than EVLA^37^. Also, the cost of care can go much higher in patients treated with EVLA, as the need for reintervention is greater in this group. The cost of care seems to vary in different regions, as some studies favor the EVLA while others favor conventional surgery^37,38^.

EVLA and conventional surgery are both equal in terms of procedural time outcome, time taken to return to normal activities or work, short-term and long-term recurrences, and clinical severity score. Differences existed in EVLA being more likely to have technical failures, needing more reintervention in the long-term, and having fewer postoperative complications. When choosing a treatment option for a patient, all these factors must be considered. Postoperative complications are less common in EVLA, but its cost and risk of the need for reintervention are the barriers that need to be assessed before choosing it over conventional surgery.

This study did not consider the intervention as per the amount of energy used in the EVLA, and this study did not consider whether USG marking was done preoperatively or not in the surgery group. Also, the procedures performed across the study were by different experts of different caliber, which may have brought heterogeneity to the studies. Another limitation of this study is that the reintervention outcome and cost of care outcome could not be included in the meta-analysis. Trials that included these parameters at their endpoints are needed to study them more promptly.

## Conclusion

EVLA and conventional surgery have no difference in short-term and long-term outcomes like procedural time, recovery time, technical failure, or clinical severity score. Technical failures were more common in the EVLA, whereas postoperative complications were more common in the surgery group. Both have comparable clinical effectiveness, and neither modality has clear superiority over the other. Other parameters, like cost-effectiveness, must be assessed at the hospital level before choosing the right procedure for the patients.

## Ethical approval

Ethical approval was not required for this systematic review and meta-analysis.

## Consent

Informed consent was not required for this systematic review and meta-analysis.

## Sources of funding

No funding received.

## Author contribution

O.S. and S.B.: contributed to conceptualization of the study and protocol development; O.S., N.T., and S.K.: contributed to the literature search and screening; N.T., S.K., P.P., and S.P.: contributed to data extraction; O.S. and S.B.: performed analysis and interpreted the data; O.S., N.T., S.K., P.P., and S.P.: drafted the initial version of the manuscript; S.B.: revised the manuscript intellectually. All authors were involved in drafting and revising the manuscript and approved the final version.

## Conflicts of interest disclosure

The authors declare no conflicts of interest.

## Research registration unique identifying number (UIN)

Registry of Systematic Reviews/Meta-analyses with the unique identifying number reviewregistry1583. (Available from: https://www.researchregistry.com/browse-theregistry#registryofsystematicreviewsmeta-analyses/registryofsystematicreviewsmetaanalysesdetails/6441621b06cb1c0028468e32/).

## Guarantor

Oshan Shrestha, MBBS, Nepalese Army Institute of Health Sciences, Kathmandu, Nepal.

## Provenance and peer review

Not commissioned, externally peer-reviewed.

## Data availability statement

Analyzed data is publicly available (is present within the manuscript).
